# A High-Throughput Assay for Small Molecule Destabilizers of the KRAS Oncoprotein

**DOI:** 10.1371/journal.pone.0103836

**Published:** 2014-08-05

**Authors:** Joseph Carver, Thomas S. Dexheimer, Dennis Hsu, Meng-Tzu Weng, Jordan L. Smith, Rajarshi Guha, Ajit Jadhav, Anton Simeonov, Ji Luo

**Affiliations:** 1 Laboratory of Cancer Biology and Genetics, Center for Cancer Research, National Cancer Institute, Bethesda, Maryland, United States of America; 2 Division of Preclinical Innovation, National Institutes of Health Chemical Genomics Center, National Center for Advancing Translational Sciences, Bethesda, Maryland, United States of America; Institute of Human Virology, Baltimore, United States of America

## Abstract

Mutations in the Ras family of small GTPases, particularly KRAS, occur at high frequencies in cancer and represent a major unmet therapeutic need due to the lack of effective targeted therapies. Past efforts directed at inhibiting the activity of the Ras oncoprotein have proved difficult. We propose an alternative approach to target Ras by eliminating Ras protein from cells with pharmacological means. In this study, we developed a cell-based, high-content screening platform to identify small molecules that could promote the degradation of the KRAS oncoprotein. We generated an EGFP-KRAS^G12V^ fluorescence reporter system and implemented it for automated screening in 1536-well plates using high-throughput cellular imaging. We screened a library of clinically relevant compounds at wide dose range and identified Ponatinib and AMG-47a as two candidate compounds that selectively reduced the levels of EGFP-KRAS^G12V^ protein but did not affect EGFP protein in cells. This proof-of-principle study demonstrates that it is feasible to use a high-throughput screen to identify compounds that promote the degradation of the Ras oncoprotein as a new approach to target Ras.

## Introduction

Ras is a small GTPase that lies at the heart of numerous cellular signaling pathways governing growth, survival, and motility [1], [2]. Growth factor receptors activate Ras through Ras guanine nucleotide exchange factors (RasGEFs) that stimulate GTP loading on Ras. This leads to a conformational change that exposes the effector binding domain on Ras, which consequently activates downstream pathways including the MAP kinase (MAPK) pathway, the PI 3-kinase (PI3K) pathway, the small GTPases Rho, Rac and Rals, and PLCε. Ras GTPase activating proteins (RasGAPs) bind to Ras and stimulate its GTP hydrolysis to return Ras to the inactive, GDP-bound state [2]. In humans there are three Ras genes: *KRAS*, *HRAS*, and *NRAS*. Oncogenic mutations in all three Ras family members have been identified in human cancers. In particular, *KRAS* is one of the most frequently mutated oncogenes across cancer types: *KRAS* mutations occur in approximately 60–70% of pancreatic cancers, 30% of colorectal and biliary cancers, and 20% of lung and ovarian cancers [1]–[3]. The activating mutation in Ras proteins is often a point mutation in codon 12 or 13 near its GTP binding pocket, which prevents RasGAP proteins from activating the GTPase activity of Ras. Consequently, the mutant Ras protein is stuck in its GTP-bound state and constitutively signals to its downstream targets, and drives aberrant cell proliferation and survival [2], [4].

Ras mutant cancers present a class of “recalcitrant cancer” with urgent and unmet therapeutic need due to the large number of patients afflicted and the lack of effective targeted therapies [5]. Significant efforts have been devoted to targeting the Ras oncoprotein in the past two decades with only limited success. Because Ras has picomolar affinity for GTP [6], it is difficult to target it with GTP-competitive molecules analogous to ATP-competitive kinase inhibitors. The search for small molecules that could stimulate GTP hydrolysis of mutant Ras have also not been fruitful. Farnesyltransferase inhibitors, which were designed to block C-terminal farnesylation of Ras proteins and thus their membrane localization, are ineffective against KRAS because KRAS can be membrane targeted through geranylgeranylation [2]. Recent effort to inhibit KRAS localization has shifted towards inhibiting the farnesyl tail-mediated binding between KRAS and PDEδ, which is necessary for the localization of KRAS [7], but the efficacy of this new approach has yet to be established. Aside from its guanine nucleotide binding pocket, Ras lacks deep, “druggable” pockets, and its interaction with downstream effectors is mediated through relatively flat protein-protein interaction surfaces. Recent fragment-based compound screens have identified molecules that can bind to KRAS and inhibit its GTP loading by the RasGEF protein SOS [8], [9]. Small molecules that covalently interact with the mutant cysteine residue in the common KRAS^G12C^ mutant have also been found to disrupt GTP-binding and impair KRAS-BRAF association [9], [10]. It remains a challenge, however, to evolve these compounds into high-affinity, cell permeable inhibitors of KRAS.

These previous efforts at targeting the KRAS oncoprotein focused on inhibiting KRAS function. Instead, we here propose that an alternative approach is to eliminate KRAS protein from the cancer cell. Knockdown of KRAS by siRNAs and shRNAs have shown strong, selective toxicity in KRAS mutant cells, thus providing genetic validation for this approach [11]–[13]. Although siRNAs are being actively explored as a therapeutic modality, delivering siRNAs effectively to tumors *in vivo* remains a major challenge [14]. Degradation of a target protein can also be facilitated by small molecules and by peptides. One approach is to use a bivalent molecule designed to bind both the protein target and a ubiquitin ligase simultaneously, and this tethering is often sufficient to drive protein degradation [15]. In breast and prostate cancers, estradiol and dihydroxytestosterone have been coupled to a peptide ligand for the VHL E3 ligase to drive the degradation of estrogen receptor-α and androgen receptor, respectively [16]. For KRAS, it has been shown that the expression of a fusion polypeptide consisting of the Ras-binding domain of CRAF and an E3 ligase is sufficient to drive KRAS degradation [17]. There is also precedence that monovalent small molecules can also trigger protein degradation. Arsenic trioxide, which is used to treat acute promyelocytic leukemia, binds directly to the PML-RARα oncoprotein and promotes its degradation through a SUMO-mediated pathway [18]. Hsp90 inhibitors indirectly reduce the levels of oncogenic proteins such as MYC and HER2 that are client proteins of the Hsp90 chaperone [19], [20]. These precedents indicate that it might be possible to identify small molecules that can trigger the unfolding and degradation of KRAS protein.

In this study, we developed an image-based high-throughput screen that can be used to search for small molecules that promote the loss of the KRAS^G12V^ oncoprotein. We demonstrate that our assay is robust and sensitive, and can be easily automated for 1536-well screens. As a proof of principle, we screened 465 mechanistically well-annotated, clinically relevant compounds and identified Ponatinib and AMG-47a as candidate molecules that could potentially impact the stability of the KRAS oncoprotein.

## Materials and Methods

### Cell culture

293T, HeLa, HeLa EGFP-KRAS^G12V^, HeLa EGFP-KRAS^WT^ and HeLa EGFP cells were cultured in Dulbecco's Modified Eagle's Medium (DMEM, Lonza, Walkersville, MD) supplemented with 10% heat inactivated fetal bovine serum (Gibco, Life Technologies, Grand Island, NY) and 100 units/mL penicillin plus 100 µg/ml streptomycin (Lonza, Walkersville, MD). The colorectal cancer cell line SW620 was from Dr. Thomas Ried [21] and cultured in McCoy's 5A medium with L-glutamine (Lonza, Walkersville, MD) supplemented with 10% heat inactivated fetal bovine serum (Gibco, Life Technologies, Grand Island, NY) and 100 units/mL penicillin plus 100 ug/ml streptomycin (Lonza, Walkersville, MD). All cells were maintained at 37°C and 5% CO_2_.

### Plasmid construction and generation of reporter cell lines

EGFP-KRAS^G12V^ DNA and EGFP-KRAS^WT^ cDNA were cloned downstream of the tetracycline response element between the Age I and Mlu I restriction sites in the pINDUCER-10b lentiviral vector, a derivative of the pINDUCER-10 vector [22] with the shRNA cassette removed. The control EGFP vector was cloned into pInducer-10b in a similar fashion using Age I and Not I restriction sites. Plasmids were packaged using 293T cells with *Trans*IT-293 Transfection Reagent (Mirus Bio, Madison, WI) according to the manufacturer's protocol. HeLa cells were transduced with pInducer EGFP-KRAS^G12V^, EGFP-KRAS^WT^ and EGFP viruses at a low MOI in media containing 1 µg/mL polybrene. Stably transduced cells were seeded as single cells in 96-well plates and selected with 3 µg/mL puromycin for 3 days. Individual clones were tested with 100 ng/mL doxycycline for protein induction. Clones with high EGFP-KRAS^G12V^, EGFP-KRAS^WT^ or EGFP expression were first selected visually using fluorescence microscopy, and GFP fluorescence levels for the clones of each type were then determined by flow cytometry. The HeLa clone with the strongest EGFP-KRAS^G12V^ induction was expanded and used for the compound screen and all follow-up assays. Both pooled and clonal HeLa EGFP and EGFP-KRAS^WT^ cells were used for counter-screening in follow-up assays.

### Small molecule screen

The NCATS MIPES 3.0 compound library has been described recently [23]. The high-throughput screen was conducted in clear-bottom 1536-well plates (Brooks Automation, Inc., Chelmsford, MA). Twenty-four hours prior to the screen, HeLa EGFP-KRAS^G12V^ cells in log phase were induced with 500 ng/mL doxycycline. Induced cells were seeded into 1536-well plates at a density of 90 cells in 4 µL of media containing 500 ng/mL doxycycline. Uninduced HeLa EGFP-KRAS^G12V^ cells in doxycycline-free media were plated in each plate as a baseline negative control. Immediately after plating, a 16-point, two-fold dilution series of Torin-1 was transferred to the screen plates by robotic pin transfer (Kalypsys, San Diego, CA). The MIPE 3.0 library was next pin-transferred into the same plates in 11-point dilution series. After 48 hours of compound incubation, all plates were washed three times with DPBS using a Biotek EL406 Microplate Washer Dispenser (Biotek, Winooski, VT). Cells were then fixed with 4% PFA and stained with 1 µg/mL DAPI.

### Image acquisition and data analysis

For plate scanning using the Acumen ^e^X3 instrument (TTP Labtech, Melbourn, UK), DAPI signal was acquired at 405 nm with a laser power of 6 mW; FITC signal was acquired at 488 nm with a laser power of 6.5 mW. Objects were thresholded by size between 1 µm and 100 µm, and background signal thresholding was set to a sensitivity of 2 SDs.

For the IN Cell Analyzer 2000 platform (GE Healthcare Bio-Sciences, Pittsburgh, PA), each well was imaged at 10x magnification with an exposure time of 300 ms for the DAPI channel and 1700 ms for the FITC channel. TIF image files were analyzed using the IN Cell Analyzer 1000 workstation with a multi-target analysis protocol. The DAPI fluorescent channel was segmented using a top-hat algorithm and a minimum object size of 120 µm^2^ to filter out fluorescent debris and artifacts. The FITC channel was used to detect EGFP-KRAS^G12V^ and a multi-scale top-hat algorithm with a characteristic object area of 1000 µm^2^ segmented whole cell area. Cells were also filtered using a limit on the per cell gyration radius of 50 µm. From the segmented images, total cell count was calculated per well. EGFP-KRAS^G12V^ fluorescence in both nuclear and cytoplasmic bitmaps and background fluorescence were also calculated as the average per cell in each well. Average total fluorescence per cell was then calculated by subtracting background fluorescence from both the nuclear and cytoplasmic EGFP-KRAS^G12V^ fluorescence and then summing these values. The primary analysis of hits was accomplished by calculating a z-score for each well based on the mean and standard deviation of EGFP-KRAS^G12V^ fluorescence from intra-plate DMSO control wells (64 per plate). Compounds with lowest z-scores of less than −5 and a consistent dose-response effect on EGFP-KRAS^G12V^ fluorescence were then manually selected as primary hits. IC50 values were calculated with Graphpad Prism 6.0 using nonlinear regression on normalized fluorescence values and log_10_ transformed concentrations.

### Flow cytometry

HeLa EGFP-KRAS^G12V^, EGFP-KRAS^WT^ or EGFP cells were seeded at a density of 25,000 cells per well in a 24-well plate in either doxycycline media (500 ng/mL doxycycline in DMEM for HeLa EGFP-KRAS^G12V^ and EGFP-KRAS^WT^ cells; 50 ng/mL doxycycline in DMEM for HeLa EGFP cells) or media alone (DMEM). Cells were treated with varying concentrations of AMG-47a, Ponatinib, and Torin-1. Media and compound were refreshed after 3 days on cells being treated for 5 days. After treatment, cells were trypsinized and resuspended in DMEM, and immediately analyzed using a FACS Calibur instrument (Beckson-Dickinson). Similar instrument settings were used for all HeLa EGFP-KRAS^G12V^, EGFP-KRAS^WT^ and EGFP samples. Fluorescence was analyzed as the median signal for each sample and data was normalized to DMSO controls. All experiments were performed with at least three independent biological replicates.

### Western blot

Cells were lysed directly using Laemmli sample buffer, and whole cell lysates were denatured at 95°C for 10 minutes and separated on either Mini-Protean TGX 4–20% gels (Bio-Rad Laboratories Inc., Hercules, CA) or 10% polyacrylamide gels. Protein was transferred to nitrocellulose membrane (Bio-Rad), and blotted with primary antibodies to KRAS (Sigma-Aldrich, clone 4F3), phospho-ERK (Cell Signaling Technology, #4377), total ERK (Cell Signaling Technology, #9102), phospho-Akt (Cell Signaling Technology, #4058), Akt (Cell Signaling Technology, #9272), EGFP (Santa Cruz Biotechnology, #SC-8354), and GAPDH (Santa Cruz Biotechnology, #FL-335). Blots were developed using HRP conjugated anti-rabbit or anti-mouse secondary antibodies and Luminata Forte substrate (Millipore, Billerica, MA).

## Results

### Establishment of reporter cell line expressing inducible EGFP-KRAS^G12V^

**Figure 1:**
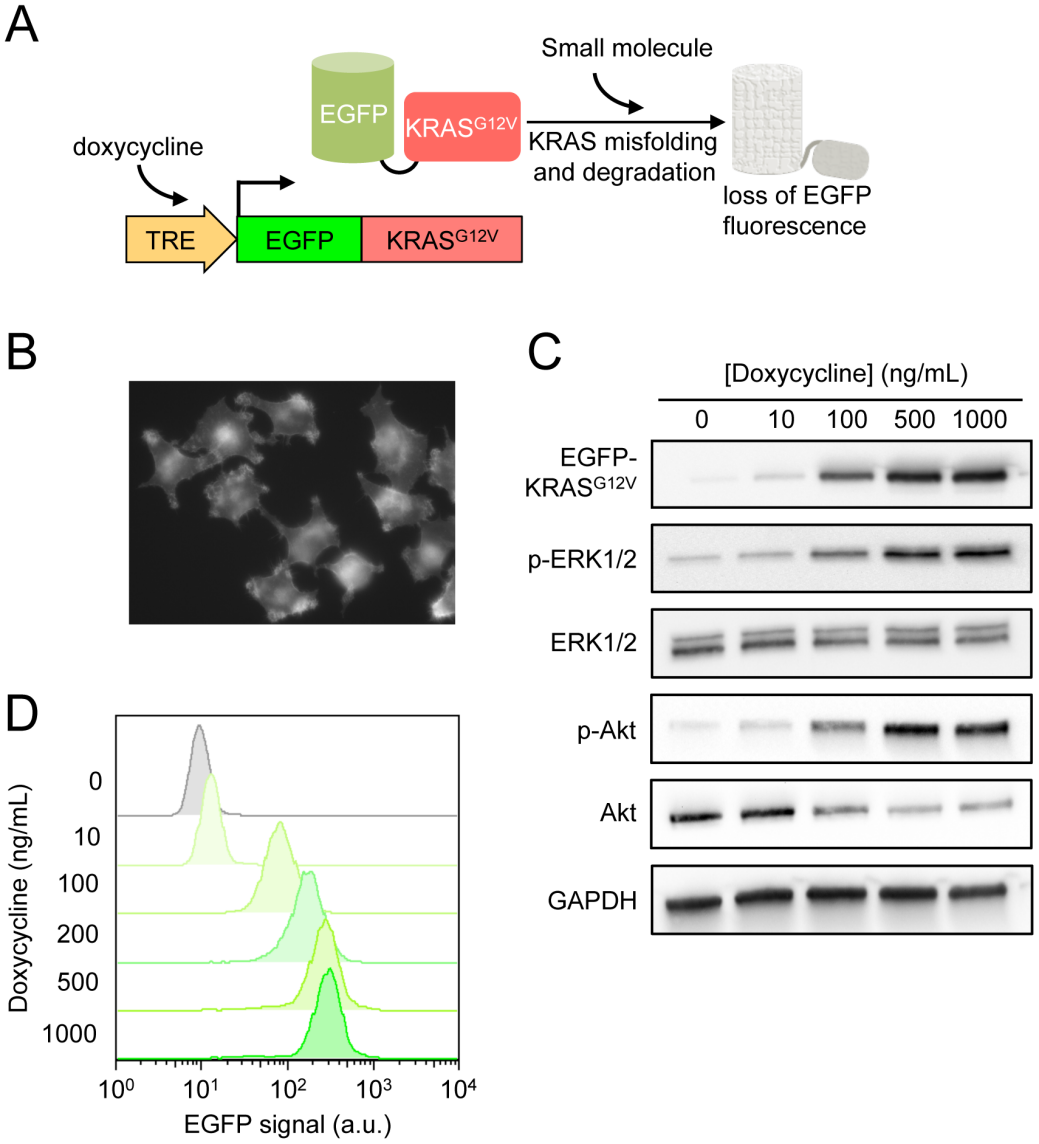
Design and validation of the EGFP-KRAS^G12V^ reporter cell line. **A.** The EGFP-KRAS^G12V^ reporter was expressed under a tetracycline-inducible promoter. Small molecules that cause the destabilization and/or degradation of EGFP-KRAS^G12V^ can be detected by measuring the changes in fluorescence signal in the cell. A control reporter expressing only EGFP from the same vector (not illustrated) was also constructed for use in counter screen. **B.** Fluorescent microscopy image of HeLa cells expressing EGFP-KRAS^G12V^ 48 hours after induction with 500 ng/mL doxycycline indicate that the fusion protein is enriched at the plasma membrane. **C.** Western blot of whole cell lysates showing the functional activation of ERK 1/2 and Akt, as measured by their phosphorylation, in HeLa cells following EGFP-KRAS^G12V^ induction. The induction level of EGFP-KRAS^G12V^ approached maximum at 500 ng/mL doxycycline. **D.** Flow cytometry quantification of dose-response induction of EGFP-KRAS^G12V^ in HeLa cells 48 hours after doxycycline. The fluorescence signal of EGFP-KRAS^G12V^ approached maximum at 500 ng/mL doxycycline.

To establish a cell-based reporter for KRAS protein expression that is independent of promoter activity, we constructed a pInducer-based lentiviral vector [22] that expresses a fusion protein consisting of the human KRAS^G12V^ mutant protein with an N-terminal EGFP tag under the control of a doxycycline-inducible promoter (Figure 1A). We used an inducible system because chronic over-expression of KRAS^G12V^ in most cell lines appeared to be toxic and was subject to strong negative selection. We transduced this inducible EGFP-KRAS^G12V^ construct into HeLa cells and selected single cell clones with stable vector integration. We next used flow cytometry to measure inducible EGFP-KRAS^G12V^ expression in these clones, and identified a HeLa clone with the highest inducible EGFP signal for further development of the screen. The EGFP-KRAS^G12V^ fusion protein is localized to the plasma membrane (Figure 1B), indicating that this reporter is processed correctly for membrane targeting. In western blot, we observed strong, doxycycline-dependent expression of EGFP-KRAS^G12V^ and activation of the MAPK and PI3K pathway as indicated by phosphorylation of ERK and Akt (Figure 1C). Thus the fusion protein is functionally intact and is able to activate its cognate downstream effectors. Doxycycline titration identified a maximal doxycycline dose at 1 µg/mL as judged by a plateau in EGFP-KRAS^G12V^ signal (Figure 1D). We chose 500 ng/mL of doxycycline for EGFP-KRAS^G12V^ induction for the screen to obtain high EGFP-KRAS^G12V^ expression without incurring toxicity.

### Optimization of high-content screening protocol

**Figure 2:**
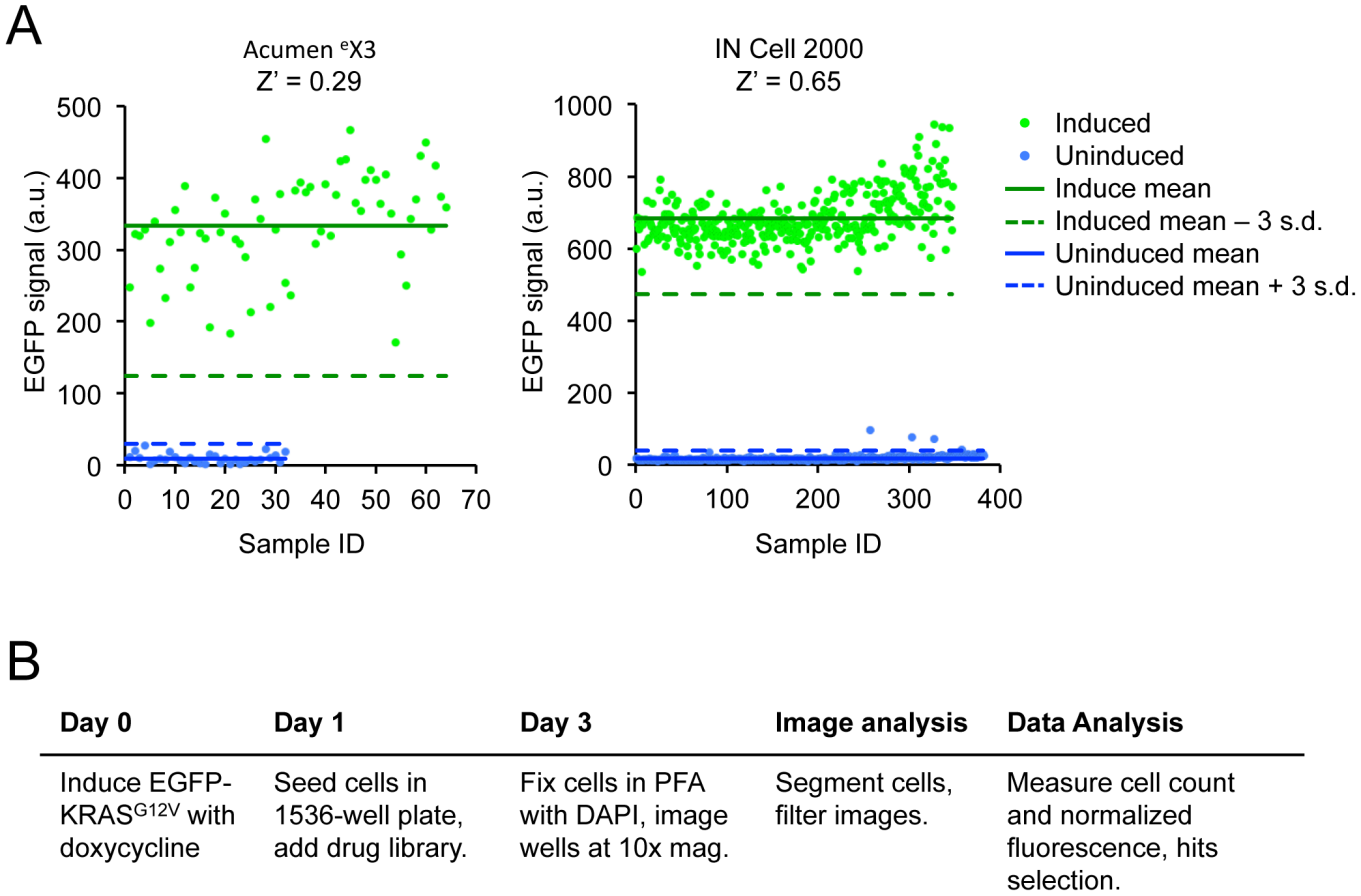
Optimization of screen parameters in 1536-well plates. **A.** Comparison of signal and background from wells containing induced and uninduced EGFP-KRAS^G12V^ cells, respectively, as measured by the Acumen ^e^X3 microplate cytometer (left) and the IN Cell Aanalyzer 2000 high-content imaging platform (right). Within each platform the data was normalized using the associated software for the calculation of Z′ score. Each data point represents a single well. **B.** Optimized workflow for the primary screening and data analysis using the IN Cell Analyzer 2000 platform.

We first adapted the EGFP-KRAS^G12V^ cell line for high-throughput screening in 1536-well plate format. The EGFP-KRAS^G12V^ reporter level was relatively low in cells even under optimal induction conditions, thus requiring a sensitive instrument for detection. In order to maximize EGFP signal collection and enable data normalization based on cell numbers, cells in 1536-well plate were fixed, permeabilized and stained with DAPI to identify their nuclei. We first compared the Acumen ^e^X3 microplate cytometer and the IN Cell Aanalyzer 2000 high-content imaging platform for detecting EGFP signal of induced and uninduced EGFP-KRAS^G12V^ cells in 1536-well plate. The data from the IN Cell Aanalyzer 2000 yielded a significantly higher signal-to-background ratio, likely due to its ability to subtract local background from the fluorescence of delineated cell objects (Figure 2A). We thus further optimized our screening and image collection protocols on the IN Cell Aanalyzer 2000 by reducing the doxycycline-induction time prior to compound treatment and by acquiring images at 10x magnification. With this platform, our primary readout is the background-subtracted average single-cell EGFP signal. We could routinely achieve Z′ factors >0.65 between wells with or without EGFP-KRAS^G12V^ induction (Figure 2A), indicating this assay is appropriate for high-throughput screening. In addition, because the raw data were stored as images, our assay has the potential to identify compounds that disrupt the membrane localization of EGFP-KRAS^G12V^ with the appropriate image analysis.

In the optimized screening protocol (Figure 2B), HeLa EGFP-KRAS^G12V^ cells were pre-treated with 500 ng/mL doxycycline for 24 hours. Cells were then plated in 1536-well plates at 90 cells/well in doxycycline-containing media, and the small molecule library was immediately added by pin transfer. Two days after compound addition, cells were fixed, stained with DAPI, and imaged. Images were subsequently analyzed with the IN Cell software.

### Screening a clinically active compound library for EGFP-KRAS^V12^ destabilizers

Using the optimized screening protocol, we screened the National Center for Advancing Translational Sciences (NCATS) MIPE 3.0 compound library comprised of 465 highly annotated small molecules, many of which are either FDA approved or in clinical development [23]. MIPE 3.0 contains a significant number of kinase inhibitors and each compound in the library was arrayed with a full-range, 11 dose-point dilution series to enable quantitative measurement of dose-dependent activity in the primary screen.

**Figure 3:**
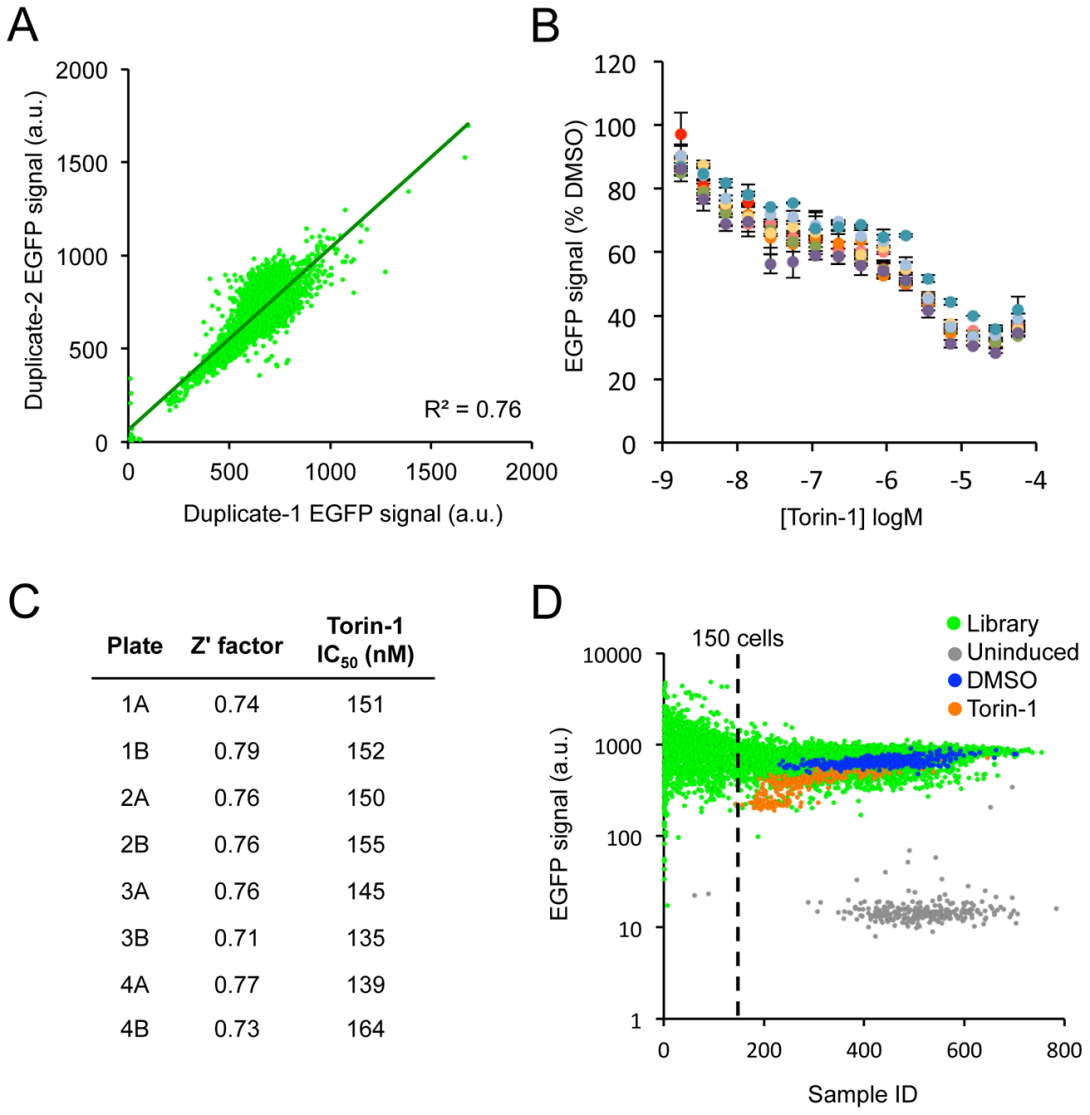
Assessment of the sensitivity and reproducibility of the primary screen. **A.** Correlation of corresponding wells from duplicate plates in the library. Wells with fewer than 150 cells were excluded from this and all subsequent analysis. Regression line and associated R^2^ value are shown. **B.** Dose-response curves of Torin-1 treated wells from all plates showing the high reproducibility of its activity in the screen (error bars represent SD from all Torin-1 wells within each plate). **C.** Z′ factors and Torin-1 IC_50_ values of all library plates (A and B are duplicates plates). **D.** Graph of EGFP-KRAS^G12V^ fluorescence signal and cell count of all wells in the library. Data series are colored to show the distribution of different control wells. Wells with fewer than 150 cells were excluded from the analysis due to high EGFP signal variation at very low cell density and for the purpose of filtering out compound doses that are overtly toxic.

During assay development we identified Torin-1, an ATP-competitive inhibitor of mTOR [24], as a potent inhibitor of EGFP-KRAS^G12V^ fluorescence. As mTOR inhibition is known to reduce overall protein synthesis [24], [25], this is likely a non-specific means to decrease EGFP-KRAS^V12^ levels in cell (see below), but nevertheless it could serve as a useful control. We screened the MIPE 3.0 library in duplicate with DMSO as a negative control and Torin-1 as a positive control. Signal correlation between duplicate plates was high (Figure 3A). Torin-1 dose curves from each library plate showed highly reproducible inhibition activity (Figure 3B), and both the Z′ factors (between induced and uninduced wells) and the 50% maximum inhibition values (IC50) for Torin-1 were consistent across multiple plates (Figure 3C). Thus we concluded that the screen was both robust and reproducible.

**Figure 4:**
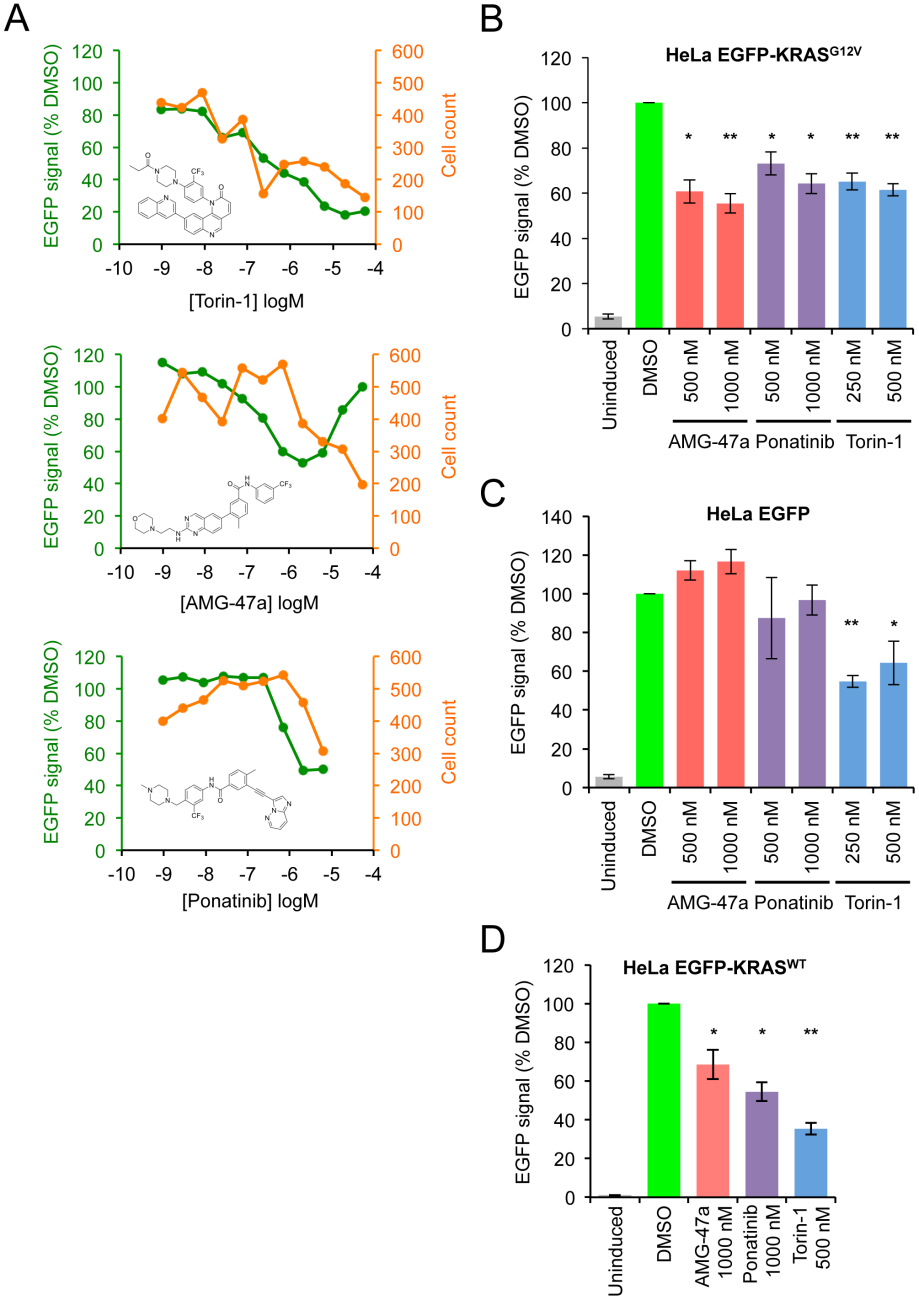
Validation of hit compounds by flow cytometry. **A.** Mean EGFP-KRAS^G12V^ signal and cell count from wells treated with Torin-1, AMG-47a and Ponatinib from the primary screen. **B–D** Flow cytometry quantification of HeLa cells expressing either EGFP-KRAS^G12V^ (B), EGFP (C) or EGFP-KRAS^WT^ after 48 hours exposure to compounds. (*p<0.05 and **p<0.001, two-tailed Student's t-test. Error bars represent SEM of at least three independent experiments).

To identify active compounds in the screen, we first normalized the EGFP signal to intra-plate DMSO control wells. We next filtered out wells that had fewer than 150 cells in order to exclude toxic compound concentrations. 150 cells represents approximately 34% of the average cell number in DMSO-treated wells, and we noticed that below this threshold the EGFP signals were more variable (Figure 3D), likely due to the loss of accuracy in the image analysis software's ability to measure per-cell fluorescence at very low cell density. We ranked the remaining wells according to the z-scores of their fluorescence, with more negative z-scores indicating greater loss of fluorescence. Hit compounds were chosen based on having strong maximal inhibition with z-score <−5 at their highest non-toxic concentration, and having a dose-dependent effect on the EGFP-KRAS^G12V^ signal. As expected, Torin-1 scored very strongly by these criteria (Figure 3B). Including Torin-1, we identified 18 candidate hit compounds of diverse known activities, including MEK, BRAF, and a variety of RTK inhibitors as potential hits (Figure 4A and Figure S3).

Because the MIPE 3.0 library contains multiple inhibitors that share common protein targets, we could potentially assess whether the activity of a hit compound was related to its intended target. In addition to Torin-1, there were 7 other mTOR inhibitors in the library. Only Torin-1 decreased EGFP-KRAS^G12V^ signal whereas the others did not, despite they all having similar cytotoxic profiles (Figure S1A). Similarly, among the 6 ABL kinase inhibitors in the library, only Ponatinib and two other compounds reduced EGFP-KRAS^G12V^ signal appreciably (Figure S1B). Thus, it is possible that the activity of Torin-1 and Ponatinib in this assay could be due to polypharmacology beyond the inhibition of their cognate protein targets.

### Secondary analysis of hit compounds AMG-47a and Ponatinib

Two of the strongest hits were Ponatinib, a pan BCR-ABL kinase inhibitor [26], and AMG-47a, a potential Lck kinase inhibitor. In the primary screen these compounds decreased EGFP-KRAS^G12V^ signal by ∼40% at 1 µM (Figure 4A). Concentrations of AMG-47a above 1 uM increased EGFP-KRAS^G12V^ signal, possibly because of higher concentrations of this compound lead to more apoptotic cells with higher autofluorescence. We thus decided to move forward with doses of AMG-47a and Ponatinib at near the maximally effective concentrations in validation assays. We first confirmed that these compounds decreased fluorescence signal in the HeLa EGFP-KRAS^G12V^ cells by flow cytometry. A 48-hour treatment of cells by AMG-47a and Ponatinib led to a 30–40% decrease in EGFP signal in these cells (Figure 4B & Figure S2A); treating cells for 3 and 5 days yielded similar results (Figure S2B & S2C). To test for the selectivity of these compounds, we generated HeLa cells expressing EGFP from the same inducible vector as controls and used these cells in a counter-screen. We reasoned that the HeLa EGFP cells would be sensitive to compounds that show non-specific inhibitory activities against the doxycycline-inducible promoter, against general RNA transcription and protein translation, or against the fluorescence or stability of EGFP. Using flow cytometry, we observed that Torin-1 indeed decreased the fluorescence signal in the HeLa EGFP cells, likely through its inhibition of general protein translation. On the other hand, Ponatinib and AMG-47a did not affect EGFP levels in these cells (Figure 4C & Figure S2). We further assessed the effect of Ponatinib, AMG-47a, and Torin-1 on the fluorescence of HeLa cells expressing EGFP-KRAS^WT^ and found that they also decreased EGFP-KRAS^WT^ signal (Figure 4D). Thus these compounds do not appear to discriminate between mutant and WT KRAS proteins.

**Figure 5:**
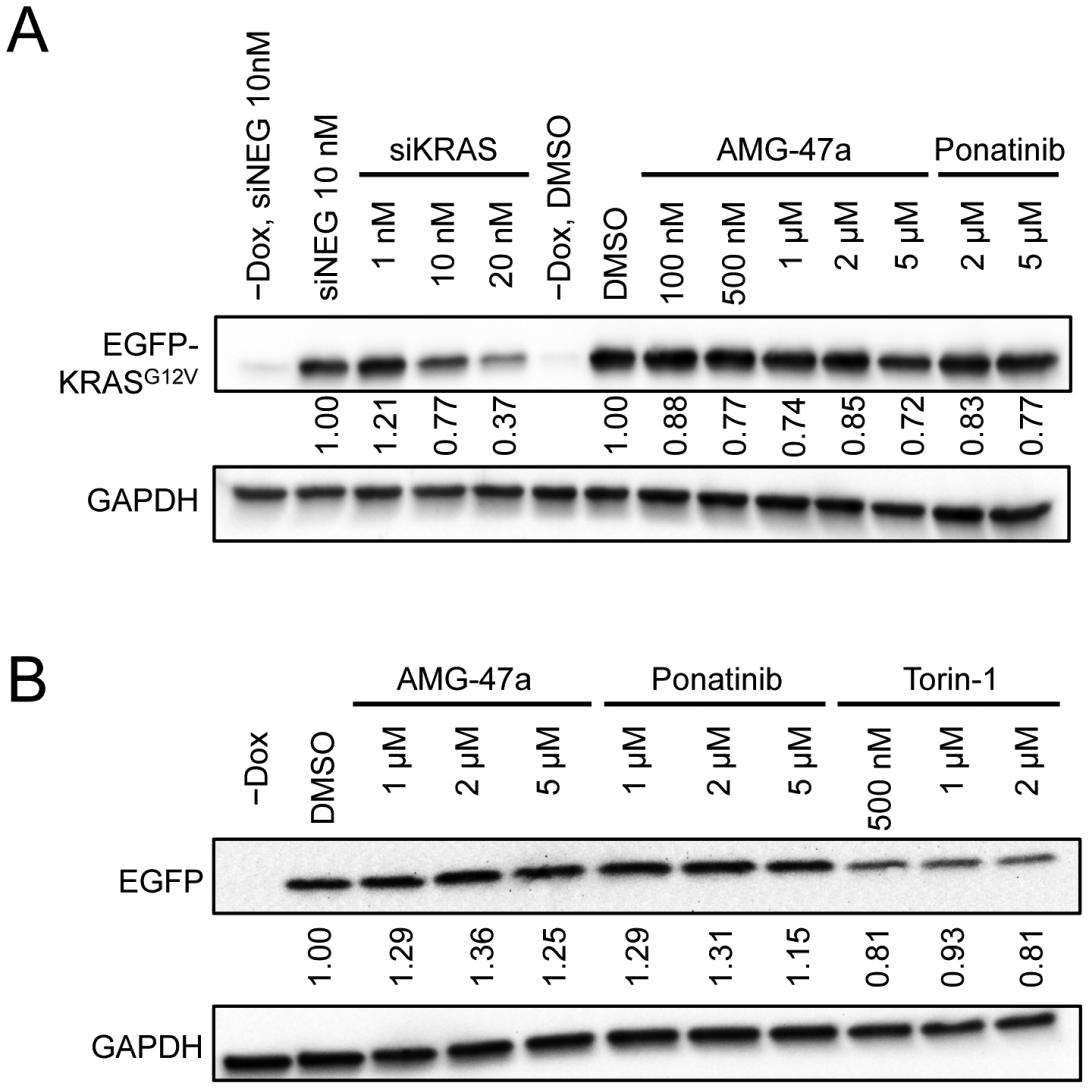
Validation of hit compounds by western blot. **A.** Levels of EGFP-KRAS^G12V^ protein in HeLa cells treated with a KRAS siRNA (siKRAS), AMG-47a and Ponatinib were probed with KRAS antibody in whole cell lysates. Numbers below the blot indicated relative protein levels. The siKRAS treated samples were normalized to siNEG control, and the compound treated samples were normalized to DMSO control. **B.** Levels of EGFP protein in HeLa cells treated with AMG-47a, Ponatinib and Torin-1 were probed with EGFP antibody in whole cell lysates. Numbers below the blot indicate relative protein levels normalized to DMSO control.

Lastly, we tested the loss of EGFP-KRAS^G12V^ proteins directly by western blot. As a positive control, we transfected a KRAS siRNA into HeLa EGFP-KRAS^G12V^ cells and observed a dose-dependent reduction in EGFP-KRAS^G12V^ protein levels. Both AMG-47a and Ponatinib had a modest effect on EGFP-KRAS^G12V^ protein levels after 3 days (Figure 5A). In the primary screen, the loss of EGFP-KRAS^G12V^ signal plateaued at ∼50% for AMG-47a and ∼55% for Ponatinib, though we were only able to consistently detect a 20–30% reduction in western blot protein levels. Although this decrease was small, both compounds had no effect on the levels of the control EGFP protein (Figure 5B). Together these results support the notion that AMG-47a and Ponatinib selectively affect the levels of EGFP-KRAS^G12V^ protein in the cell.

## Discussion

In this study we developed a high-throughput cell-based assay that uses an inducible EGFP-KRAS^G12V^ reporter to identify small molecules that affect the stability of the KRAS oncoprotein. We screened a library of clinically relevant compounds and identified 18 candidates that diminished the EGFP-KRAS^G12V^ fluorescence signal by up to 50% at doses that are not overtly cytotoxic. Among these are the mTOR kinase inhibitor Torin-1, the ABL kinase inhibitor Ponatinib and the Lck kinase inhibitor AMG-47a. Torin-1 has the strongest activity in the library, but its action is non-selective as it also reduced the fluorescence signal of cells expressing only EGFP. The ability of Torin-1 to block overall protein translation could be a partial explanation. However, we noted that none of the seven other mTOR inhibitors in the library were effective at reducing EGFP-KRAS^G12V^ fluorescence, despite having similar cytotoxicity profiles. Ponatinib, one of the strong hits in the screen, has been primarily described as an inhibitor of BCR-ABL kinase [26]. The screen identified two other BCR-ABL inhibitors – Nilotinib and DCC-2036 – as hits, though three other ABL inhibitors in the library did not display any activity. Thus it is unlikely that the inhibition of ABL alone can account for the loss of EGFP-KRAS^G12V^ in Ponatinib-treated cells. The polypharmacology of Torin-1 and Ponatinib could therefore be useful in re-purposing them as starting scaffolds for new pharmacological properties. While there is no obvious connection between kinase inhibition and KRAS oncoprotein levels, staurosporines, which binds to the ATP-binding pockets of many kinases, have been shown to relocalize KRAS^G12V^ to cellular endosomes and drive its proteasome-independent degradation [27]. Though none of the staurosporine analogues in the MIPE 3.0 library showed activity in our screen, it is possible that the short assay duration of 48 hours in our primary screen precluded us from detecting the activity of these compounds.

The composition of the MIPE 3.0 library was enriched for clinically relevant oncology drugs that are either FDA approved or in clinical trials, many of which are ATP-competitive kinase inhibitors. We do not expect this small library to contain molecules that potently drive EGFP-KRAS^G12V^ degradation. The activity of Ponatinib, AMG-47a and other hits was accordingly modest in the primary screen. Measurement of fluorescence signal, both on the IN Cell platform and by FACS, indicates that Ponatinib and AMG-47a compounds reduced EGFP-KRAS^G12V^ level by ∼40% consistently without affecting the levels of EGFP, although this difference was more modest when measured by western blot, which was less sensitive and less quantitative. In SW620 colorectal cancer cells which express endogenous mutant KRAS, Ponatinib and AMG-47a were highly toxic at concentrations required to observe loss of EGFP-KRAS^G12V^ in HeLa cells (>1 µM), thus we were unable to test whether these compounds also affect the levels of endogenous KRAS oncoprotein. Further screening of a larger library with more diverse chemical structures would be necessary to identify compounds with better activity profiles.

Because Ras proteins are relatively stable and no specific ubiquitin ligases have been attributed to the degradation of Ras, the mechanism of its turnover is not well understood [28], [29]. Ras rapidly undergoes large structural oscillations between the active and inactive conformations [30], thus its folding is highly dynamic and there might be opportunities for small molecules to bind to Ras and trigger its unfolding or mis-folding, which in turn could lead to its degradation by the cellular protein quality control pathway [31]. Our screen thus serves as a proof-of-principle to demonstrate the feasibility of using an EGFP-KRAS^G12V^ reporter assay in a high-throughput format to identify small molecules that could drive Ras degradation. In addition, this assay could potentially enable the identification of compounds that disrupt the membrane localization of full-length KRAS protein when cells are imaged at sufficiently high resolutions [32], [33]. For proteins such as Ras – in which the total protein level and its subcellular localization both affect signaling output and cellular phenotype – this assay could be a valuable approach to high-throughput screening.

## Supporting Information

Figure S1 **Comparison of small molecules with similar mode of action in the screen.** **A.** Dose-dependent effects of 8 mTOR inhibitors on EGFP-KRAS^G12V^ signal and cell number in the primary screen. Only Torin-1 had a significant effect on EGFP-KRAS^G12V^ signal. **B.** Dose-dependent effects of 6 ABL kinase inhibitors on EGFP-KRAS^G12V^ signal and cell number in the primary screen. Only Ponatinib, Nilotinib and DCC-2036 had a significant effect on EGFP-KRAS^G12V^ signal.
(TIF)

Figure S2 **Effect of hit compounds on EGFP-KRAS^G12V^ and EGFP fluorescence signal.** **A.** Fluorescent and phase contrast microscopy images of HeLa cells expressing either EGFP-KRAS^G12V^ or EGFP with or without compound treatment for 48 hours. **B. & C.** Flow cytometry quantification of HeLa cells expressing either EGFP-KRAS^G12V^ (top) or EGFP control (bottom) after either 3 days (B) or 5 days (C) of exposure to compounds. (*p<0.05 and **p<0.001, two-tailed Student's t-test. Error bars represent SEM of three independent experiments).
(TIF)

Figure S3 **Additional hit compounds from the primary screen.** Mean EGFP-KRAS^G12V^ signal and cell count from wells treated with compounds at indicated concentrations are shown. Reported primary target for each compound is given below its name.
(TIF)

## References

[1] Karnoub AE, Weinberg RA (2008) Ras oncogenes: split personalities. Nat Rev Mol Cell Biol 9: 517–531 10.1038/nrm2438 18568040 PMC3915522

[2] Cox AD, Der CJ (2010) Ras history: The saga continues. Small GTPases 1: 2–27 10.4161/sgtp.1.1.12178 21686117 PMC3109476

[3] Pylayeva-Gupta Y, Grabocka E, Bar-Sagi D (2011) RAS oncogenes: weaving a tumorigenic web. Nat Rev Cancer 11: 761–774 10.1038/nrc3106 21993244 PMC3632399

[4] Malumbres M, Barbacid M (2003) RAS oncogenes: the first 30 years. Nat Rev Cancer 3: 459–465 10.1038/nrc1097 12778136

[5] Stephen AG, Esposito D, Bagni RK, McCormick F (2014) Dragging Ras Back in the Ring. Cancer Cell 25: 272–281. 24651010 10.1016/j.ccr.2014.02.017

[6] John J, Sohmen R, Feuerstein J, Linke R, Wittinghofer A, et al (1990) Kinetics of interaction of nucleotides with nucleotide-free H-ras p21. Biochemistry 29: 6058–6065. 2200519 10.1021/bi00477a025

[7] Zimmermann G, Papke B, Ismail S, Vartak N, Chandra A, et al. (2013) Small molecule inhibition of the KRAS-PDEδ interaction impairs oncogenic KRAS signalling. Nature: 1–5. doi:10.1038/nature12205. 10.1038/nature12205 23698361

[8] Maurer T, Garrenton LS, Oh A, Pitts K, Anderson DJ, et al (2012) Small-molecule ligands bind to a distinct pocket in Ras and inhibit SOS-mediated nucleotide exchange activity. Proc Natl Acad Sci USA 109: 5299–5304 10.1073/pnas.1116510109 22431598 PMC3325706

[9] Sun Q, Burke JP, Phan J, Burns MC, Olejniczak ET, et al (2012) Discovery of small molecules that bind to K-Ras and inhibit Sos-mediated activation. Angew Chem Int Ed Engl 51: 6140–6143 10.1002/anie.201201358 22566140 PMC3620661

[10] Ostrem JM, Peters U, Sos ML, Wells JA, Shokat KM (2013) KRAS(G12C) inhibitors allosterically control GTP affinity and effector interactions. Nature: 1–14. doi:10.1038/nature12796. 10.1038/nature12796 PMC4274051 24256730

[11] Wee S, Jagani Z, Xiang K, Loo A, Dorsch M, et al. (2009) PI3K Pathway Activation Mediates Resistance to MEK Inhibitors in KRAS Mutant Cancers. Cancer Research. doi:10.1158/0008-5472.CAN-08-4765. 10.1158/0008-5472.CAN-08-4765 19401449

[12] Sunaga N, Shames DS, Girard L, Peyton M, Larsen JE, et al (2011) Knockdown of oncogenic KRAS in non-small cell lung cancers suppresses tumor growth and sensitizes tumor cells to targeted therapy. Molecular Cancer Therapeutics 10: 336–346 10.1158/1535-7163.MCT-10-0750 21306997 PMC3061393

[13] Zorde Khvalevsky E, Gabai R, Rachmut IH, Horwitz E, Brunschwig Z, et al. (2013) Mutant KRAS is a druggable target for pancreatic cancer. Proc Natl Acad Sci USA. doi:10.1073/pnas.1314307110. 10.1073/pnas.1314307110 PMC3870687 24297898

[14] Kanasty R, Dorkin JR, Vegas A, Anderson D (2013) Delivery materials for siRNA therapeutics. Nat Mater 12: 967–977 10.1038/nmat3765 24150415

[15] Raina K, Crews CM (2010) Chemical inducers of targeted protein degradation. J Biol Chem 285: 11057–11060 10.1074/jbc.R109.078105 20147751 PMC2856979

[16] Rodriguez-Gonzalez A, Cyrus K, Salcius M, Kim K, Crews CM, et al (2008) Targeting steroid hormone receptors for ubiquitination and degradation in breast and prostate cancer. Oncogene 27: 7201–7211 10.1038/onc.2008.320 18794799 PMC5573236

[17] Ma Y, Gu Y, Zhang Q, Han Y, Yu S, et al (2013) Targeted degradation of KRAS by an engineered ubiquitin ligase suppresses pancreatic cancer cell growth in vitro and in vivo. Molecular Cancer Therapeutics 12: 286–294 10.1158/1535-7163.MCT-12-0650 23288781

[18] Zhang X-W, Yan X-J, Zhou Z-R, Yang F-F, Wu Z-Y, et al (2010) Arsenic trioxide controls the fate of the PML-RARalpha oncoprotein by directly binding PML. Science 328: 240–243 10.1126/science.1183424 20378816

[19] Regan PL, Jacobs J, Wang G, Torres J, Edo R, et al (2011) Hsp90 inhibition increases p53 expression and destabilizes MYCN and MYC in neuroblastoma. Int J Oncol 38: 105–112. 21109931 PMC3212671

[20] Friedland JC, Smith DL, Sang J, Acquaviva J, He S, et al (2014) Targeted inhibition of Hsp90 by ganetespib is effective across a broad spectrum of breast cancer subtypes. Invest New Drugs 32: 14–24 10.1007/s10637-013-9971-6 23686707 PMC3913847

[21] Grade M, Hummon AB, Camps J, Emons G, Spitzner M, et al (2011) A genomic strategy for the functional validation of colorectal cancer genes identifies potential therapeutic targets. Int J Cancer 128: 1069–1079 10.1002/ijc.25453 20473941 PMC3008507

[22] Meerbrey KL, Hu G, Kessler JD, Roarty K, Li MZ, et al (2011) The pINDUCER lentiviral toolkit for inducible RNA interference in vitro and in vivo. Proc Natl Acad Sci USA 108: 3665–3670 10.1073/pnas.1019736108 21307310 PMC3048138

[23] Mathews Griner LA, Guha R, Shinn P, Young RM, Keller JM, et al (2014) High-throughput combinatorial screening identifies drugs that cooperate with ibrutinib to kill activated B-cell-like diffuse large B-cell lymphoma cells. Proc Natl Acad Sci USA 111: 2349–2354 10.1073/pnas.1311846111 24469833 PMC3926026

[24] Thoreen CC, Chantranupong L, Keys HR, Wang T, Gray NS, et al (2012) A unifying model for mTORC1-mediated regulation of mRNA translation. Nature 485: 109–113 10.1038/nature11083 22552098 PMC3347774

[25] Huo Y, Iadevaia V, Yao Z, Kelly I, Cosulich S, et al (2012) Stable isotope-labelling analysis of the impact of inhibition of the mammalian target of rapamycin on protein synthesis. Biochem J 444: 141–151 10.1042/BJ20112107 22428559

[26] O'Hare T, Shakespeare WC, Zhu X, Eide CA, Rivera VM, et al (2009) AP24534, a pan-BCR-ABL inhibitor for chronic myeloid leukemia, potently inhibits the T315I mutant and overcomes mutation-based resistance. Cancer Cell 16: 401–412 10.1016/j.ccr.2009.09.028 19878872 PMC2804470

[27] Cho K-J, Park J-H, Piggott AM, Salim AA, Gorfe AA, et al (2012) Staurosporines disrupt phosphatidylserine trafficking and mislocalize Ras proteins. J Biol Chem 287: 43573–43584 10.1074/jbc.M112.424457 23124205 PMC3527944

[28] Ulsh LS, Shih TY (1984) Metabolic turnover of human c-rasH p21 protein of EJ bladder carcinoma and its normal cellular and viral homologs. Mol Cell Biol 4: 1647–1652. 6092927 10.1128/mcb.4.8.1647-1652.1984 PMC368962

[29] Ahearn IM, Haigis K, Bar-Sagi D, Philips MR (2012) Regulating the regulator: post-translational modification of RAS. Nat Rev Mol Cell Biol 13: 39–51 10.1038/nrm3255 PMC3879958 22189424

[30] Shima F, Ijiri Y, Muraoka S, Liao J, Ye M, et al. (2010) Structural basis for conformational dynamics of GTP-bound Ras protein. J Biol Chem. doi:10.1074/jbc.M110.125161. 10.1074/jbc.M110.125161 PMC2903345 20479006

[31] Sontag EM, Vonk WIM, Frydman J (2014) Sorting out the trash: the spatial nature of eukaryotic protein quality control. Current Opinion in Cell Biology 26: 139–146 10.1016/j.ceb.2013.12.006 24463332 PMC4204729

[32] Prior IA, Hancock JF (2012) Ras trafficking, localization and compartmentalized signalling. Semin Cell Dev Biol 23: 145–153 10.1016/j.semcdb.2011.09.002 21924373 PMC3378476

[33] van der Hoeven D, Cho K-J, Ma X, Chigurupati S, Parton RG, et al (2013) Fendiline inhibits K-Ras plasma membrane localization and blocks K-Ras signal transmission. Mol Cell Biol 33: 237–251 10.1128/MCB.00884-12 23129805 PMC3554123

