# Supplementary figures and images for: A High-Throughput Assay for Small Molecule Destabilizers of the KRAS Oncoprotein
Source: PLoS One. 2014 Aug 5;9(8):e103836. doi: 10.1371/journal.pone.0103836 (PMC4122376; doi:10.1371/journal.pone.0103836)

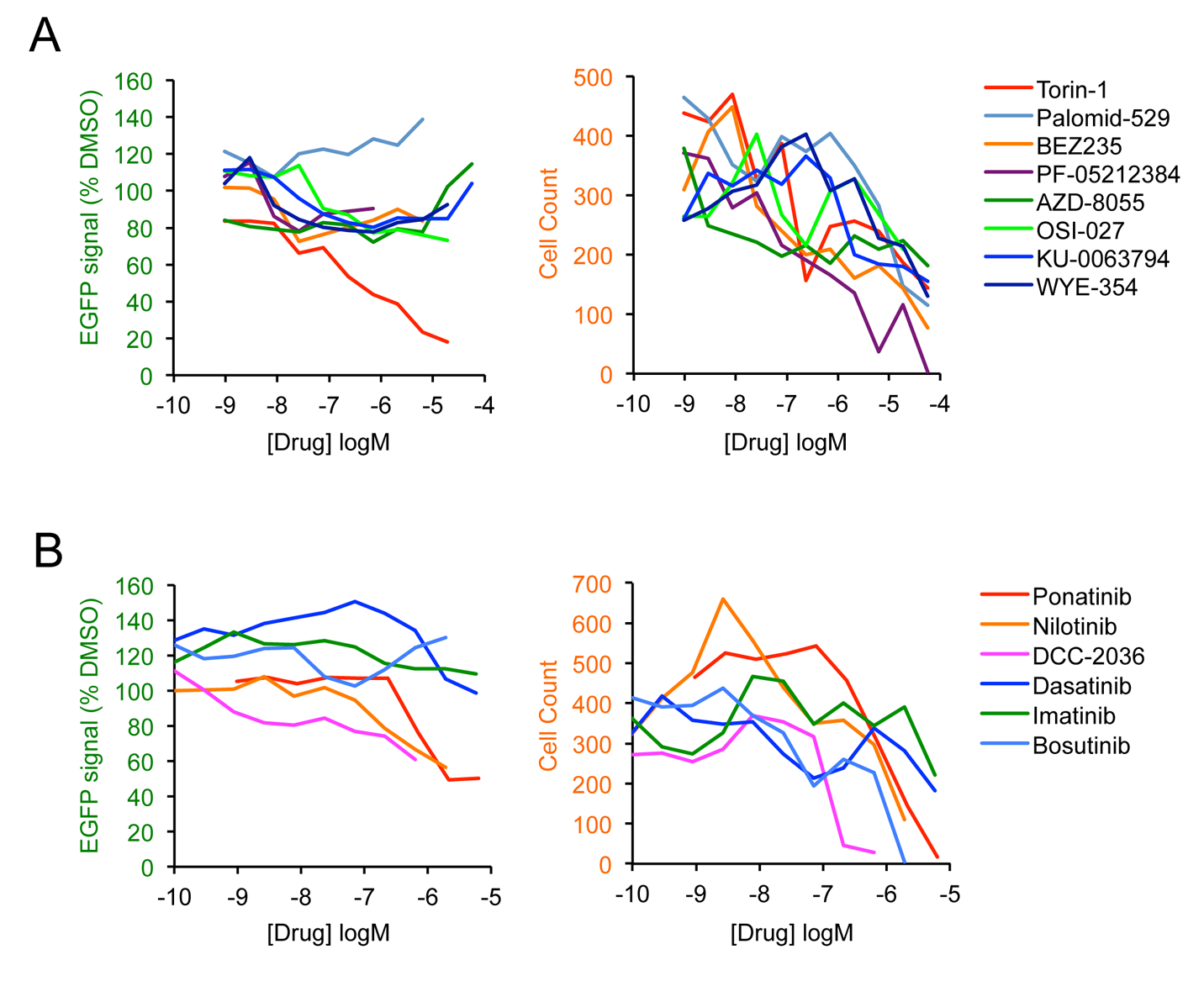

Supplement: Figure S1 — Comparison of small molecules with similar mode of action in the screen. A. Dose-dependent effects of 8 mTOR inhibitors on EGFP-KRASG12V signal and cell number in the primary screen. Only Torin-1 had a significant effect on EGFP-KRASG12V signal. B. Dose-dependent effects of 6 ABL kinase inhibitors on EGFP-KRASG12V signal and cell number in the primary screen. Only Ponatinib, Nilotinib and DCC-2036 had a significant effect on EGFP-KRASG12V signal. (TIF) [file pone.0103836.s001.tif]

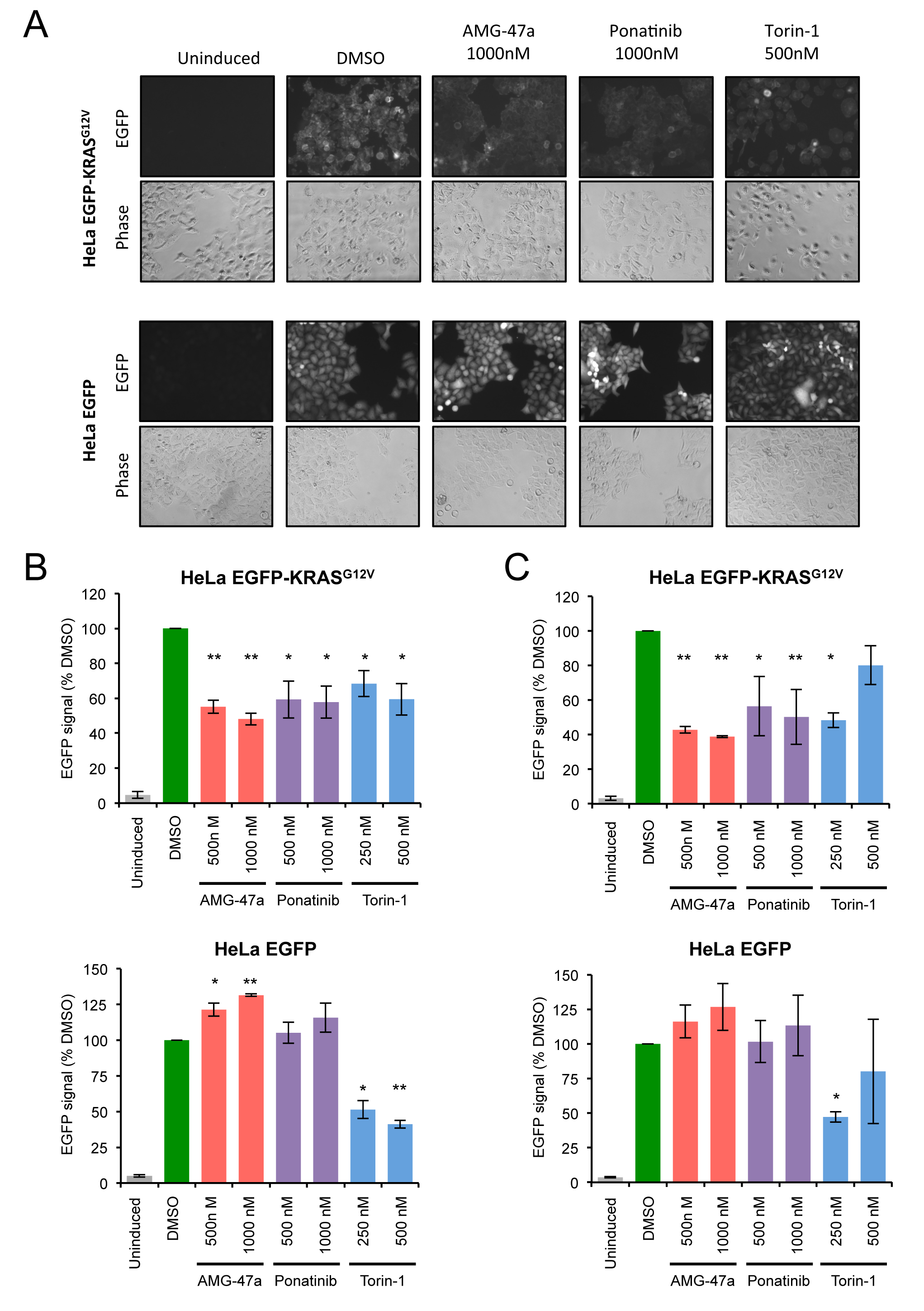

Supplement: Figure S2 — Effect of hit compounds on EGFP-KRASG12V and EGFP fluorescence signal. A. Fluorescent and phase contrast microscopy images of HeLa cells expressing either EGFP-KRASG12V or EGFP with or without compound treatment for 48 hours. B. & C. Flow cytometry quantification of HeLa cells expressing either EGFP-KRASG12V (top) or EGFP control (bottom) after either 3 days (B) or 5 days (C) of exposure to compounds. (*p<0.05 and **p<0.001, two-tailed Student's t-test. Error bars represent SEM of three independent experiments). (TIF) [file pone.0103836.s002.tif]

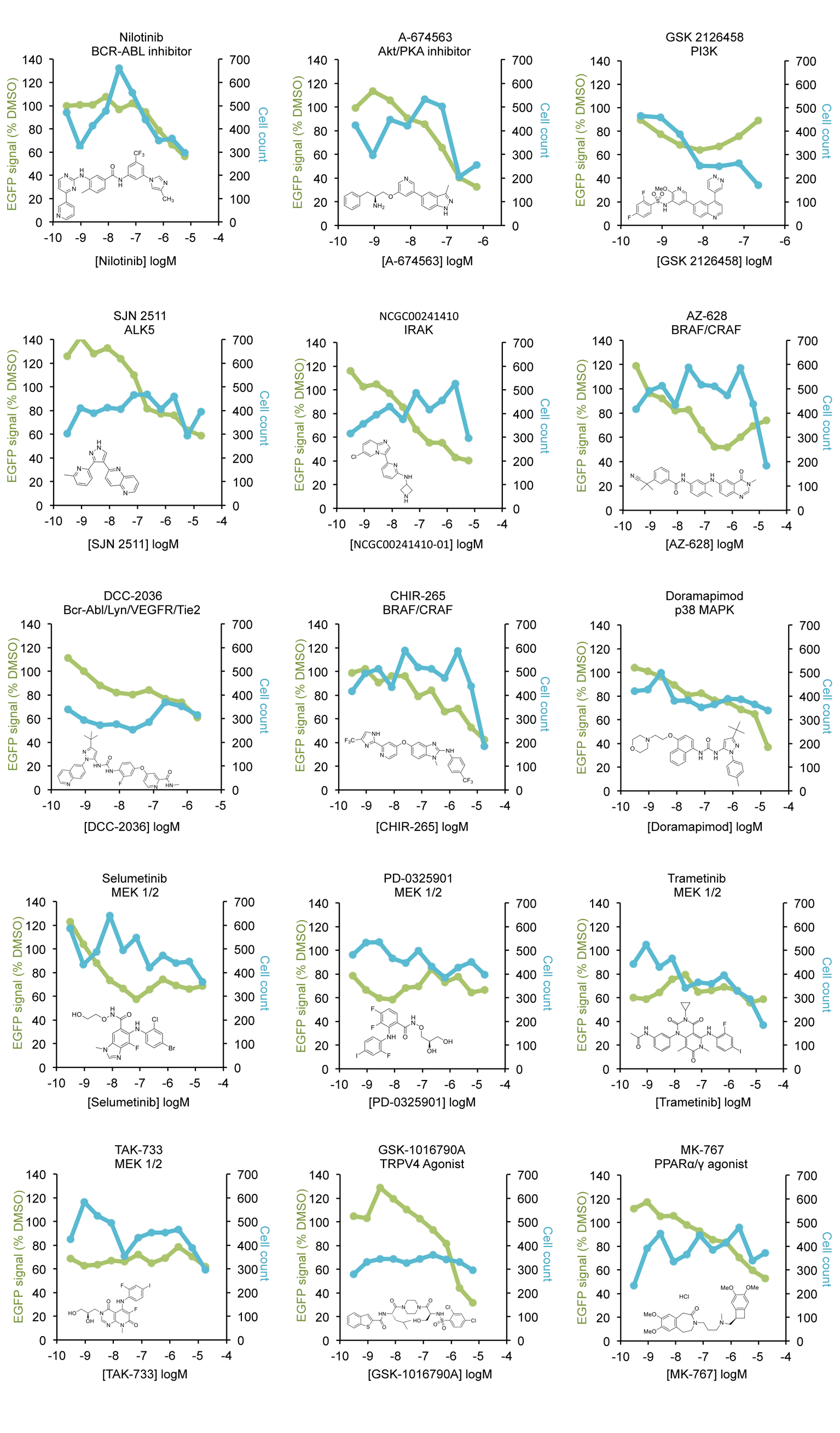

Supplement: Figure S3 — Additional hit compounds from the primary screen. Mean EGFP-KRASG12V signal and cell count from wells treated with compounds at indicated concentrations are shown. Reported primary target for each compound is given below its name. (TIF) [file pone.0103836.s003.tif]
